# From a Single Voice to Diversity: Reframing ‘Representation’ in Patient Engagement

**DOI:** 10.1177/10497323231221674

**Published:** 2024-01-16

**Authors:** Brett Scholz, Lucy Kirk, Terri Warner, Lauren O’Brien, Zsuzsoka Kecskes, Imogen Mitchell

**Affiliations:** 1 School of Medicine and Psychology, 2219 The Australian National University, Canberra, ACT, Australia; 2 ACT Disability, Aged and Carer Advocacy Service, Canberra, ACT, Australia; 3 School of Medicine, 8691 University of Wollongong, Wollongong, NSW, Australia; 4 Canberra Health Services, Canberra, ACT, Australia

**Keywords:** consumer representation, consumer leadership, consumer involvement, patient and public involvement, service user involvement, lived experience leadership

## Abstract

There has been a growing emphasis on consumer representation in the development of health policy, services, research, and education. Existing literature has critiqued how discourses of representativeness can disempower consumers working in health systems. The context of the current study is consumer engagement in the development of COVID-19 triage policy and practice in a local health service. Consumer engagement has often been an afterthought in the COVID response, with few examples of consumers in agenda-setting or decision-making roles. In the Australian Capital Territory, 26 consumer, carer, and community groups worked together with academics and clinicians to develop these principles. Interviews were conducted with stakeholders (including consumers, clinicians, and other health professionals) to evaluate the development of triage principles. A discursive psychological approach to analysis was used to explore participants’ understandings about and constructions of consumers being representative (or not) and how this may reproduce power imbalances against consumers. The results explore two distinct ways in which participants talked about consumer representativeness: the first drawing on rhetoric about consumers as lay members of the public (as distinct from being professionally engaged in the health sector), and the second in terms of consumer representatives being diverse and having intersectional identities and experiences. Expectations about consumers to be representative of the general population may reproduce traditional power imbalances and silence lived experience expertise. These power imbalances may be challenged by a shift in the way representativeness is conceptualised to requiring health services to seek out diverse and intersectionally marginalised consumers.

## Introduction

Contemporary health policies require consumer engagement in all stages and levels of planning, implementation, delivery, and evaluation across the health sector (Australian Commission on Safety and Quality in Health Care (ACSQHC), 2012; Patient Engagement Action Team, 2017). There are also several important benefits to greater involvement, including the development of better care (Longden et al., 2016), improving social justice (Scholz et al., 2017b), and providing more relevant innovation across health systems (Scholz et al., 2018b). Throughout this article, we use ‘consumer’ as it is the term most often used in the Australian health policy context (National Health and Medical Research Council, 2016). We acknowledge, though, there are existing critiques of this terminology, and in other contexts different terms would be considered more appropriate such as public and patient involvement (Ocloo et al., 2021), service user engagement (Abayneh et al., 2017), or representation of people with lived experience (Breault et al., 2018).

In some areas, there has been a strong history of advocacy driven by a need for consumers to have a say in the development or reform of services such as in mental health (Scholz et al., 2017b) or HIV/AIDS care (Roy & Cain, 2001). Meaningful, non-tokenistic engagement of consumers in health services more broadly remains limited (Scholz et al., 2018a, 2018c; Ehrlich et al., 2020). Thus, the extent to which services meet the needs or ideals of consumer engagement varies widely.

There are several barriers limiting meaningful consumer engagement in the health sector. First, working together with consumers presents a paradigm shift for many health professionals who may be threatened or feel their authority is challenged (Kopera et al., 2015), or may not understand how or why they should be collaborating so closely with consumers (Bennetts et al., 2013). Second, there are inherent power imbalances between consumers and non-consumers working in the health sector, with consumers often positioned at the bottom of organisational decision-making structures, without power (Lewis, 2014). Consumers are often left out of important discussions or are not provided with sufficient resources to enable meaningful collaboration (Clarke et al., 2023), or have to work harder than non-consumers to be seen as equals (Scholz et al., 2017a). Another barrier relates to the way consumers are positioned in their engagement roles as being ‘representative’ of a wider consumer group with little clarity about what that might mean (Moran et al., 2013). Findings from the growing body of work about these power differentials between consumers and other health professionals suggest that the health sector cannot truly benefit from consumers’ experiential expertise until it is valued to the same extent as other health professionals’ disciplinary knowledge (Scholz et al., 2018b).

The concept of ‘consumer representation’ is used differently across contexts. There is an established critique of the term specifically within the mental health context. For instance, consumers working within the mental health sector are often required to have capacity to ‘represent’ consumers as a group but at the same time have been criticised by other health professionals for being ‘unrepresentative’ of consumers *because* they have more capacity than the group is popularly assumed to have (Stewart et al., 2019). Further, it has been shown claims of representativeness (or lack thereof) are used in different ways to result in empowerment or disempowerment of consumers (Scholz et al., 2017c). Specifically, requiring individual consumers to represent *all* consumers’ perspectives serves to disempower consumers and devalue their individual contributions, while providing space for a diversity of consumers could be empowering (Scholz et al., 2017c). The argument consumers should be representative has previously been used to silence activism and question the legitimacy of consumer roles (Happell & Roper, 2006). Representativeness may even be understood by other health professionals to require some kind of democratic inclusion of consumers (Fischer & Van de Bovenkamp, 2019). Other ways that representativeness has been understood include consumer representatives being authorised by or accountable to the broader group of people with lived experience, or somehow symbolic of those they represent (Maguire & Britten, 2017).

Reconceptualising expectations about representativeness has been an emerging area within the literature for some time (see Happell & Roper, 2006, for a seminal exploration of the ‘myth’ of representation). There have been more recent calls to shift the onus from the individual consumer to *be* representative, to the organisation or system to develop diverse representation strategies in order to benefit from a more diverse group of consumers (Scholz et al., 2018a). There are benefits to ensuring there be more than one token consumer because when the power imbalance between consumers and other stakeholders is present, services are less likely to benefit from consumers’ input (Horgan et al., 2021; Scholz et al., 2020a; Scholz et al., 2018b). Further, questioning individual consumers’ ‘representativeness’ could be considered unfair given research has noted other health professionals are not usually scrutinised in the same way (Scholz et al., 2017c).

### Consumer Engagement in the Context of COVID-19

Community engagement can ensure outbreak responses are delivered in a contextually appropriate manner, but a rapid review of the published evidence of engagement in the context of disease control during epidemics found most examples of community engagement do not focus on or consider equity issues (Gilmore et al., 2020). This may be because pandemic impacts can be felt quickly and heavily in some jurisdictions whose systems were overwhelmed (Tangcharoensathien et al., 2021), limiting the time and resources available for meaningful engagement. Nonetheless, consumer organisations advocated for health services to engage with consumers even in the COVID-19 context (Health Consumers NSW, 2020).

The pandemic provided an important context for the current study for two reasons. First, existing conceptualisations of consumer representation have largely been very narrowly focused on specific health conditions or types of service use – such as mental health (Happell & Roper, 2006; Scholz et al., 2017c). The context of COVID-19 at the time meant that ‘consumers’ had to be conceptualised both quite broadly (i.e. at the time it was unclear exactly who would be impacted by COVID and it felt likely to be anyone and everyone) and specifically (i.e. it was expected that people with disabilities, older people, and Aboriginal and Torres Strait Islander people would be likely impacted in worse and specific ways). Second, despite a large number and range of consumers likely to be impacted by the pandemic, they were often excluded from decision-making about the COVID response. This context allowed us to explore how participants made sense of consumer representation when consumers more broadly had been excluded despite the concern that even more consumers were likely to be impacted than in any other recent health crisis.

The current study arose from an evaluation of the consumer engagement that led to the development of a triage process for potentially scarce critical care resources during the COVID-19 pandemic within the Australian Capital Territory (ACT). Just like in other jurisdictions in Australia (such as Queensland, Health Consumers Queensland, 2020) and internationally (e.g. British Columbia, Canada; British Columbia Ministry of Health, 2020), the COVID-19 response team in the ACT moved quickly in March 2020 to develop a framework for triage for allocation of scarce critical care healthcare resources. As a collaboration between Health Care Consumers Association ACT, the Medical School at the Australian National University, and the Australian Capital Territory COVID-19 response team, a more inclusive collaborative process was developed when it became clear the relatively low transmission rates in Australia allowed for more time for consumers, carers, and community members to be part of the production of the triage system (Scholz et al., 2020b).

### Theoretical Framework

The core principles underpinning co-production partnerships with consumers are as follows: (1) consumers are partners from the outset, (2) power differentials are explored and addressed, and (3) consumer leadership and capacity is developed (Roper et al., 2018). As a theoretical framework, the current study draws on ways in which power imbalances are central to collaborations between consumers and other health professionals to consider how particular understandings about consumer representation interact with the power differentials in co-production partnerships.

Power dynamics in such collaborative endeavours are complex. In addition to the power imbalances we have already discussed, recent research has found inequities across organisational levels that reproduce power differentials including diverse concerns such as other health professionals controlling the financing and resourcing, roles and expectations, and recruitment of consumer representatives (Ocloo et al., 2021). Recently, power has been conceptualised in research about collaborations between consumers and other health professionals as the ability of one actor to influence another (O’Shea et al., 2019). We draw on this definition but also a practical dimension of power in such collaborations – specifically, the extent to which consumers have decision-making and agenda-setting power in health systems, organisations, and initiatives (Stewart et al., 2023).

Previous research has explored ways in which rhetoric (understood within discourse analysis as text or talk used to reproduce and strengthen specific understandings of the world; Potter, 1996) of representation can disempower or empower consumers in such partnerships (Scholz et al., 2017c). Through this theoretical lens, the current study seeks to explore how power differentials are acknowledged, explored, and addressed (or not) in relation to understandings of representativeness.

### Aim and Objectives

The aim of the current study is to examine stakeholders’ (including consumers and advocates, clinicians, and academics) perceptions about how consumer ‘representativeness’ shaped the consumer engagement process. To meet this aim, the key objectives are as follows:

1. To collect data about stakeholders’ perception of consumer engagement within the context of a COVID-19 policy development process;
2. To apply the theoretical framework of power differentials in co-production to these data and focus on the ways in which stakeholders in the development of the triage process understand representativeness; and
3. To examine the relationship between perceived power and understandings of representation in collaborative processes between consumers and health systems.

## Materials and Methods

### Design

This study used a qualitative and exploratory design (Stebbins, 2001) and examines the way stakeholders understand consumer representation in the context of the collaborative development of the triage process for ICU resources during the COVID-19 pandemic.

### Data Collection

Participants (total *n* = 14) included members of the team tasked to co-produce the triage framework (a senior health executive, a senior research fellow, and a medical student and research assistant) (*n* = 3); clinicians that would use the framework (*n* = 2); and members of the 26 consumer, carer, or community organisations who were part of its development (*n* = 9). This last group included people in dedicated consumer representation roles, people in administrative roles within those organisations, and people who have had experience in representation and administration. As such, the participants come from a wide range of perspectives (including in advocacy, health services, health education, health research, and health policy); a wide range of areas of expertise (such as within the disability sector or supporting Aboriginal and Torres Strait Islander communities); and a wide range of experiences and years within the system (including CEOs of community organisations with broader relevant knowledge or members of organisations whose work focuses more deeply and specifically on advocacy). During the development of the triage framework, each organisation was invited to contact their members in a way most appropriate for them to invite them to be part of the collaboration. Some people participating in the collaboration were public members of these organisations; however, due to the timing (peri-pandemic) and sensitivity (concerns of wellbeing and safety) of the discussions, some organisations decided it would be most appropriate for executive members of the organisation to represent their constituencies. Further, some organisations were themselves systemic advocacy organisations – perhaps with additional scope to be and experience in *representing* the needs of the communities they serve.

Approval for the current study was given by the ANU Human Research Ethics Committee (#2020/272). Prior to each interview, participants were advised they could withdraw at any stage during the interview or refuse to answer any question they did not feel comfortable doing so. They were also told the sole purpose of recording the interview was to ensure accuracy of the transcript for analysis, and data would be de-identified during the transcription process. Interviews were conducted via videoconferencing. The interview questions (provided in the Supplementary Material file) were open-ended, allowing participants to direct the conversations towards issues more salient to their experiences of the collaborative process. Interviews ranged from 40 to 72 min in duration, and audio was recorded and transcribed by BS for analysis.

### Data Analysis

Data familiarisation, coding, and categorisation was undertaken by the first author who does not identify as working from a consumer perspective, but whose academic and health work is largely done in collaboration with people with lived experience. One category developed through this process contained data about how participants drew on, reproduced, and challenged the concept of representation. This was identified as an important topic for further analysis because there has been important seminal work about how representation is misunderstood (Happell & Roper, 2006) and subsequent work about how representation is discursively deployed to empower or disempower consumer representatives (Scholz et al., 2017c).

Data mapped to the category of representation were thus analysed using discursive psychological principles (Potter, 2012) which allows for more in-depth consideration of what discourse (i.e. text or talk) achieves and produces. Extracts were chosen from the data corpus related both to the category of representation and also related to power as conceptualised through the lens of co-production. In this context, the discursive psychological approach is concerned with the ways in which talk actively constructs or challenges power imbalances in consumer representation. Thus, our focus was on how participants drew on ways of understanding representation and how they constructed collaborative production of the triage system as meeting (or not) ideals of representation. All authors iteratively contributed to the analysis of the data – this included interpretation of data drawing on their experiences either as consumer representatives, consumer advocates, or other health professionals.

The discursive psychological approach does not seek to locate discourses within any individual’s cognition (e.g. it is not necessarily concerned with whether what people say is linked to their own thoughts or attitudes); rather, it suggests we draw on, reproduce, or critique existing ways of meaning-making. Thus, our approach does not vilify any individual using, for instance, what we might frame as potentially problematic or oppressive rhetoric, but rather to draw attention to what kinds of meaning-making is done through discourse. The focus of the analysis is to identify ways in which repertoires (which can be understood as the ways of communicating about something available within the public consciousness; Scholz & Stewart, 2021) inform and are informed by how we reproduce or challenge collective meaning-making in this case about the role of power dynamics in consumer representation.

## Results

In the context of the current study, stakeholders’ reflection and discussion about ‘representation’ was common. There were no primary interview questions explicitly and specifically asking participants to critique or discuss the issue of representation in and of itself, but most participants oriented to the concept of consumer representation in their interviews. There were two main ways in which participants’ accounts of *representativeness* in the collaborative process differed. Broadly, these were (a) representing the majority and (b) representation through diversity.

### Representing the Majority

A common question about consumer representation relates to whether consumers collaborating in health system work should be representing their own experiences or consumers’ collective experiences. One of the ways this question arose in the current study was through the discursive repertoire about consumers representing a broader population, as in Extract 1, from one of the health professionals involved in the development of the triage.

Extract 1

I guess I’ve always been someone who tries to work with groups of people and bring them along the journey. And I guess the hardest bit is to know how one A) does that and B) who should be within that group because I guess I’m acutely aware of the professional patient or the professional consumer voice. And I guess I’m not … I’m yet to be convinced they are representative of the majority of those that in theory that they represent. That’s what makes me feel nervous.

Of note in Extract 1 is an assumption: the desirability of being “representative of the majority.” Further, in suggesting others might need to “be convinced” consumer partners are representative of the majority, this representativeness is constructed as necessary for consumers to work within the health sector. In turn, this suggests the power of the decision about who is representative enough could lie with the decision-makers seeking representation rather than with consumers themselves. Embedded within this repertoire is the construction of the ‘professional consumer’ as an identity. Previous research has explored the paradox of discounting consumer representatives with capacity to engage in their roles because they are no longer considered representative if they have such capacity (Happell & Roper, 2006). In this extract, the juxtaposition of an identity as a “professional consumer” with a need to be “representative of the majority” is another way in which this paradox is reproduced. In this way, expectations of representativeness become linked to laity – whereby consumers who are seen as too engaged in the health sector may be no longer considered representative. The categorisation of consumer representatives as either ‘lay’ or ‘professional’ may ignore the range of expertise brought to the health sector (Reynolds & Beresford, 2020), which is concerning given this expertise needs to be valued so that health systems and organisations can benefit from it (Scholz et al., 2018b). Indeed, in suggesting it makes them “nervous” to be neglecting a majority, the participant orients to the importance of adequately highlighting all perspectives. Thus, drawing such a distinction between lay and experienced consumer representatives may represent ways in which health services have traditionally understood consumer engagement but may be unhelpful to advancing such engagements.

Another repertoire linking representation to majority concerns is through implying an individual consumer’s concerns are too specific to be representative, such as in Extract 2.

Extract 2

Well, I think it’s just trying to avoid people who’ve necessarily got a bee in the bonnet about something that may only be a bee in a bonnet for them personally rather than a bee in a bonnet for the majority and I guess it’s just trying to tease out making sure that you’ve got the broader view rather than just someone’s view because I guess just sometimes I get a bit concerned that, because one person has had this particular experience and may not be representative of other people’s experience. So I think it is trying to get as many views as possible so that you can try and land on, you know, a way forward, that’s going to be acceptable to the majority, I think in the end, you’re never gonna … well, I think it’s very hard to ensure that everyone is comfortable with whatever you’re trying to do.

In the first part of this extract, this participant draws on a common discourse about consumers having “a bee in a bonnet” (related repertoires include talking about consumers with a particular “axe to grind” or similar; Griffiths et al., 2004; Happell, 2010). The implication here being consumers with specific concerns are sometimes viewed as not being able to represent consumers more broadly. Such constructions of specific concerns as unrepresentative could be seen as potentially silencing these consumers’ perspectives (i.e. if particular concerns are only concerning a small group, then they are unhelpful). Further, an alternative repertoire could suggest addressing the “bee in a bonnet” of a particularly vocal consumer is likely to help others as well (if others’ views are also addressed), or significant changes occur in systems because of people who are vocal with their agendas. Further, such rhetoric may at times be used to construct systemic issues as personal grievances – with a risk that potentially serious concerns become trivialised. Indeed, service industry research discusses how consumer complaints are incredibly valuable (Barlow & Møller, 1996) and being able to redress complaints should be valued.

The participant in Extract 2 also then offers a potential solution to the “bee in a bonnet” dilemma, by suggesting ensuring “as many views as possible” is sought to represent consumers’ perspectives. Thus, a slight shift is hinted at, from placing the onus on individual consumers for representing beyond their specific concern to placing the onus on health systems to ensure more than one consumer perspective is sought. Given the rhetorical situation of this extract (contrasting specific gripes to solutions that are “acceptable to the majority”), the implication is still that consumers’ individual concerns are unlikely to reflect broader concerns within the community.

The tension between different understandings of representativeness is expanded upon in Extract 3 as a notable example of a participant expanding on multiple types of representation and stakeholders.

Extract 3

Q: So when you’re talking about representative samples, What might you be looking for? And do you mean representative of society more broadly, or representative of particular groups that are impacted the most by the outcome?

A: So I think it’s a really good question. And ideally, I’ve seen both. So I’ve previously seen consultation where they’ve done the random community consultation, then they had one session where they hand-selected a group that were … representative roughly of the demographics, age distribution, education of the community. And they did another one which was stakeholders. Now, when they said stakeholders, they meant people who are most likely to be impacted by the triage and primarily they actually meant healthcare workers. Now you could run this differently and put people who are actually more likely to be sickest. I think when I mean representative, I do mean generalisability to the broader community. Because that seems to be a big issue in stakeholder stuff I’ve already read that small sample size means that unfortunately we can’t generalise this to the rest of the population. And there’s participation bias and that sort of thing. So I guess I do mean representation of the of the community that you’re referring to, because that that is important. But I think there is huge value also specifically going to vulnerable population groups who might be impacted by a policy like this.

On one hand, representation is constructed as desirable to work towards being able to “generalise … to the rest of the population.” However, where Extract 1 linked generalisability with laity, Extract 3 links generalisability to numbers and specifically to the “sample size” of people working together. In some ways, this rhetoric about generalisability and sample size mirrors positivist understandings about generalisability as stemming from just one kind (statistical-probabilistic) of generalisability. This relates to what has been termed ‘statistical’ representation (Maguire & Britten, 2017) with health services withholding decision-making or agenda-setting power from consumer representatives because they lack statistical representativeness (Scholz et al., 2017c). Other forms of generalisability are less often considered within health systems but include intersectional generalisability (which is concerned with patterns across oppressed peoples and communities) which is alluded to in this extract as “specifically going to vulnerable population groups.” It is perhaps telling, though, the only generalisability referred to as ‘generalisability’ in these interviews relates to statistical-probabilistic forms of generalisability, as this is the most commonly available repertoire of generalisability (Smith, 2018). Nonetheless, the part of the rationale for the collaborative process was to ensure traditionally marginalised perspectives were amplified (Scholz et al., 2020b), and thus the “huge value” in trying to address the needs of those impacted the most was prioritised in this process.

### Representation Through Diversity

In Extracts 2 and 3, there were suggestions of a shift from representativeness as linked specifically to the majority, towards understandings of representativeness linked to inclusion of diverse perspectives. This corresponds to a shifting power dynamic whereby consumers are not excluded from agenda-setting capacity because they are deemed not representative enough. In Extract 4, for instance, this is exemplified by a participant who directly links the concept of being ‘representative’ to the “diversity of the group.”

Extract 4

I’m not sure of the ways that the opportunity was advertised to consult and the ways that you came up with the group that you approached in the end. I don’t know if some of that advertising was or wasn’t inclusive in the way that the advertising was structured and set out and in terms of the representative aspect of things … whether there’s a diversity of the group that you were consulting with, whether there was enough lived experience of a really wide variety of types, and also whether there was enough options for other [input] – I’m just thinking even academics with [lived and academic] expertise in these arenas. I know that I’d fed back early on that the research the lit review that have done it was done was from quite a – understandably – but was from a very medically focused approach and that I’d felt that it was missing a whole lot of the grey literature and the disability rights sort of lens and that having involvement by researchers with expertise in that aspect of the arena would have been of assistance. Likewise also for older people … having experts with expertise in specifically the rights of older people in this sort of space. But there’s so many different types of diversity and there’s so much intersectional experience. I’m conscious that coming up with the perfect way to approach it, it’s a complicated thing to do, particularly on topics that are and can be very traumatizing.

In referring to some of the key groups impacted by the collaborative work (namely people with disabilities or older people), and relevant intersectional identities, representativeness becomes constructed as something achievable only through wide inclusion. In such a perspective, representativeness becomes less about any individual and more about seeking out diverse, relevant representation. The onus to *be* representative, as discussed in Extracts 1–3 is instead shifted to the onus to *seek* representation, through noting the “ways that the opportunity was advertised … [to be] inclusive” was rhetorically positioned as leading to the “representative aspect” of the collaboration.

Of note in this extract is how the participant suggested “academics with expertise in” the rights of particular marginalised groups should be part of the representation of such groups. It should be noted there are parallel calls for embedding into universities more dedicated roles for lived experience academics (Classen et al., 2021). Valuing this lived experience (and privileging consumer voices over those of other stakeholders) as an important element of such expertise would mean seeking to include both consumer and carer lived experience academics where possible.

Related to this issue, one of the objectives of the original collaborative process was to try to ensure consumers, carers, and community groups had a space to develop the triage principles without having others speak ‘for’ them (Scholz et al., 2020b), but there is precedence for independent allies (i.e. those not from those minority groups but who use their privilege/power to make space for them) to advocate for and amplify the needs of these people (Juntanamalaga et al., 2019). Further, multiple participants highlighted allies are often in a position to use their power to redress power imbalances – and requiring people with lived experience of discrimination to be part of conversations about their rights (and with implications for life and death) could be traumatising (particularly in the context of videoconferencing meetings during COVID lockdowns).

The participant in Extract 5 further elaborates on the lack of intersectionality in representation:

Extract 5

I guess the other kind of observation I’d make is: at different stages across the development of the guidelines and not just from when [a more collaborative approach was adopted] is that there were probably groups missing in terms of intersectionality, so you know, Aboriginal and or Torres Strait Islander people, people who are obese, who are elderly, who are in the range of frames that were potentially at risk in terms of a triage approach whose voices weren’t always evident in the discussions. Yeah, and that’s partly because those groups are underrepresented within formal bodies, but also because it wasn’t seen as affecting them in the beginning.

The participant in this extract first mentions some of the intersectional identities of those likely to be impacted significantly in the allocation of healthcare in the context of COVID-19. This is particularly relevant to the way the collaborative process was planned (i.e. through asking peak bodies and other organisations to invite their members to contribute) and in the way these intersectionally marginalised groups and individuals “are underrepresented within formal bodies.” Positioning such marginalised people as having inadequate representation touches on the challenges and broader issues of seeking a range of people to participate. In the collaborative approach evaluated, 26 consumer, carer, and community organisations were approached to invite their membership to take part in the development of triage principles for COVID-19 (Scholz et al., 2020b). While these 26 organisations were broad and diverse, the participant in Extract 5 identifies those particularly marginalised across their intersectional identities are not always well represented in such organisations. Even in some of the invited organisations that have representation in their remit (such as the ACT Aboriginal and Torres Strait Islander Elected Body, for example, which is elected to provide “a strong democratically elected voice”; Blackburn, 2014, p. 125), such intersectional representation may not always be present.

Despite the limitations, and given the importance of seeking diverse perspectives, a broad group of stakeholders took part in the collaborative development of triage principles. One of the stakeholder groups was carers, acknowledging the important role carers play in health. In Extract 6, a participant expands upon the ongoing concerns about carers speaking *for* consumers.

Extract 6

In having the conversations moving forward, I think you would need to think about … “How do we engage carers in a conversation in a way that’s respectful to the consumer?”. Because that’s a challenge – “how do we help carers in understanding those decisions, develop a framework for decision making, even if they don’t know what the answer is.” I remember [clinician] saying to me at some point, “you know what would you want to happen if it was your mother” and … I know what my mother would think about that, but I don’t know what my brother would think about it, and that was a really poignant personal moment of reflection of going, “why is that, why is it that I have a good sense of what I think my mother would want, but not my brother” and “how do I now go and have that conversation with my brother” and I’m neither of their carers, I’m just their next of kin. So it might be about rather than asking me to as a carer to make decisions about how that triage would happen. Because I don’t know that they can tell you that. I think what would be useful, is going, “how would we as a clinical staff help you make the decision with us about what to do.”

The participant orients to broader repertoires of understanding about engaging with carers without wanting to co-opt consumers’ experiential expertise but allowing space for carers’ perspectives (about both consumers’ and carers’ needs) to be considered. This begins with prioritising ways of engaging carers while being “respectful to the consumer.” The speaker then provides an example of carer engagement at the individual service level about a specific potential interaction where an individual carer might need to make a decision about an individual consumer. Previous research has explored ways in which carers cannot and should not speak *for* consumers in systemic- and organisational-level engagement (Goodwin & Happell, 2006). Nonetheless, carers’ perspectives are important in the context of COVID-19, but it is also important carers’ perspectives are considered given their role in healthcare more broadly (e.g. what carers might need to know about “how … clinical staff [can help] make the decision with [carers] about what to do” or how carers might feel being exposed to trauma by the use of such triage tools).

## Discussion

The findings of the current study highlight ways in which different understandings of consumer representation have implications for the kinds of representation sought. Specifically, constructing representativeness as emerging from the laity of representatives (such as in Extract 1 which suggests the “professional consumer voice” is not representative) may serve to exclude consumer representatives from agenda-setting and decision-making power within the health sector. Indeed, the dichotomy between laity and professionalism has been criticised for flattening nuance of complex and changing identities of consumer representatives (Reynolds & Beresford, 2020). Similarly, constructions of representativeness as a consequence of statistical-probabilistic generalisability (such as in Extract 4 which suggests being able to “generalise [to the] population” should be the goal of engaging with consumer representatives) may lead to ignoring specific experiences directly relevant to the project at hand which would benefit from lived experience leadership in decision-making. Such understandings of representation were challenged by positioning diversity as the goal of representativeness (rather than focussing on specific consumer representativeness) and by shifting responsibility for representation to those seeking consumer representatives (to ensure they seek “enough lived experience of a really wide variety of types,” as in Extract 4). Building on previous work finding the rhetoric of representation is used in different contexts to either empower or disempower consumer representatives (Scholz et al., 2017c), the current study describes specific kinds of constructions of representativeness more likely to be empowering (i.e. those emphasising representation through diversity rather than those putting the onus to be statistically representative on any individual).

Grounding our findings within the framework of co-production acts as a reminder ‘representation’ itself is not the end goal but is rather a means to ensuring decision-making and agenda-setting in health systems is held more equitably. We note that this study explores how a triage process was built in the context of a global pandemic, and thus, perspectives of multiple groups and individuals were required. Having managed such broad inclusion in the COVID-19 context, we believe there are learnings that health systems could apply beyond the pandemic to continue to work towards greater inclusion. Thus, there are several practical implications of our findings.

First, there needs to be clarity about expectations of representativeness (and the implications of these expectations) in partnerships between consumers, health systems, and other stakeholders. For instance, expecting consumer partners to represent the general population (in a statistical-probabilistic way) such as discussed in Extract 1 is potentially neither possible nor desirable. Further, from the perspective of the theoretical framework of co-production, non-consumers policing the extent to which consumers are representative reproduces traditional power differentials; in turn, the core principle of addressing power differentials in co-production would remain unmet (Roper et al., 2018). Extending on recent research that critiques the assumption that consumer organisations are inherently ‘representative’ in a democratic way (Fischer & Van de Bovenkamp, 2019), the current study contributes to conceptualising the complexity of representation. Further, while previous research suggests questioning individual consumers’ ‘representativeness’ is problematic as other health professionals are not usually scrutinised in the same way (Scholz et al., 2017c), the current findings extend on this by suggesting a diversity of consumers helps address concerns about representation. To refine the analogy: although health professionals may not be scrutinised in the same way, there are interprofessional tensions whereby, for instance, an oncology nurse would be challenged if they claimed to represent mental health nurses. Similarly, not all consumers are the same, nor can one consumer represent all consumers. There is a tension here, though, in that we call for shifting the onus of responsibility to the health system to ensure it seeks diversity in representation. The nuance is that health systems should make space for a diversity of views to be engaged, while deliberate action needs to be taken to ensure that questioning *individual* consumer partners’ representativeness does not silence their perspectives.

Our findings thus provide further detail to the question posed by Clarke et al. (2023) about how initiatives might include a wide range of service users, voices, or perspectives. Existing research has been used to argue that it is “fruitless to blame patient groups for failing to live up to [nebulous and shifting] criteria of representativeness” (Maguire & Britten, 2017, p. 68). Our findings make a contribution to this argument by calling for collaboration with consumers to make systems more accountable in addressing consumer representation. More frank discussions between these groups about what representation means and what kinds of representation are needed will allow the operationalisation of ‘representation’ to evolve in partnership.

Second, moving towards collaborations with consumers with intersectional experiences of adverse outcomes and inequity can help health organisations with “dismantling systems of oppression” (and COVID-19 highlighted how health systems reproduced inequities; Young et al., 2020, p. 1229). Ensuring partnerships are inclusive (“specifically going to vulnerable population groups who might be impacted,” Extract 3) and diverse (ensuring there are few “groups missing in terms of intersectionality,” Extract 5) allows policy makers to mitigate the impacts of entrenched inequity. Health organisations are critiqued for often engaging only with consumers who are “younger, more likely to benefit from care, non-smokers, with dependents, healthcare workers, and those with no physical or intellectual disability” (Norman et al., 2021, p. 326). Indeed, it was because of the advocacy and experience held across the 26 organisations included in the collaborative production of the principles underlying the triage process for COVID-19 in the ACT (from which the current study was produced); the final triage was based primarily on human rights principles (Scholz et al., 2020b). If such an outcome is possible even in the context of the pandemic, then surely collaborations with more time and resources can continue to work towards better inclusion and diversity. We would add, though, there is also a corresponding responsibility to provide supports to mitigate negative impacts of engaging with such processes. For instance, in the context of the development of these triage principles, peak and advocacy bodies were asked to provide support for the consumers or members they nominated to be part of the process. This was thought to be most appropriate given the diversity of needs and the existing relationships consumers had with their own organisations. However, every collaboration is likely to have different needs, and it would be important to be flexible about what support is needed.

Third, systems should be equipped to engage with forms of generalisability beyond the traditional statistical-probabilistic model. Indeed, trying to collaborate with a sample representative of society more broadly (rather than representative of those most impacted by implementation) could be considered gerrymandering of collaborations (Kukull & Ganguli, 2012). Such an approach may bias results and lower external validity – in turn leading to poorer generalisability. Seeking out consumer collaboration with those most likely to have an adverse outcome or experience injustice (even if it is in small, statistically ‘unrepresentative’ numbers) is desirable to increase the external validity of results. In Extract 2, the participant talked about being concerned about the difficulty of ensuring “everyone [would be] comfortable” with the outcome of a collaborative project. Organisations should empower staff to collaborate with those most impacted by a given issue (e.g. in the context of COVID triage systems, older people, people with disabilities, and Aboriginal and Torres Strait Islander people were among those most likely to be impacted by unfair systems), so they can feel confident in how collaborating with consumers with specific experiences actually increases the external validity of the development and implementation of services.

A potential limitation of the current study is participants were drawn from those people who were present in the collaborative production of the principles of the triage process. As such, there are likely people who were not ‘represented’ in collaboration (and particularly those who are intersectionally oppressed) who should have been whose views have not been included in our analysis. Although there was broad consumer, carer, and community engagement (Scholz et al., 2020b), people from extremely vulnerable groups (including, notably, homeless or incarcerated people) were neither included in the collaborative processes nor the current study. Although the current study has contributed to understandings of representation in collaborations and partnerships with consumers, it would be helpful for future work to consider the myriad power relations implicated in the ways in which representation is sought.

## Conclusion

Although consumer engagement in the context of COVID-19 was often an afterthought while health organisations struggled to keep up with the need for services across many jurisdictions, some examples (such as the development of principles underlying triage for COVID-19 in the ACT) have allowed us to explore perceptions of consumer representation in pandemic response. Conceptualisations of representation in terms of laity of consumers or appealing to statistical generalisability appear to reproduce power imbalances against consumers in collaborations with the rest of the health sector. However, conceptualisations of representation as it relates to seeking intersectional and experiential diversity take the onus off of individual consumers to be somehow representative and place it on health systems to ensure they are seeking and adequately supporting diverse engagement.

## Supplemental Material

Supplemental Material - From a Single Voice to Diversity: Reframing ‘Representation’ in Patient Engagement Supplemental Material for From a Single Voice to Diversity: Reframing ‘Representation’ in Patient Engagement by Brett Scholz, Lucy Kirk, Terri Warner, Lauren O’Brien, Zsuzsoka Kecskes, and Imogen Mitchell in Qualitative Health Research
