# Supplementary material for: From a Single Voice to Diversity: Reframing ‘Representation’ in Patient Engagement
Source: Qual Health Res. 2024 Jan 16;34(11):1007–18. doi: 10.1177/10497323231221674 (PMC11487870; doi:10.1177/10497323231221674)
Supplement: Supplemental Material - From a Single Voice to Diversity: Reframing ‘Representation’ in Patient Engagement [file sj-pdf-1-qhr-10.1177_10497323231221674.pdf]

Supplementary Material: Interview Schedule

Interviews were semi-structured, allowing for participants to discuss topics and experiences relevant to them. Follow-up questions and prompts were given depending on participants' responses, but the primary questions asked of all participants were:

1. In what ways did you observe the inclusion of consumers/carers/community members having an impact on the development of the framework?

2. How were the challenges of involvement overcome?

3. Did the involvement process allow everyone to have a say?

4. Whose voices were silenced despite the consumer/carer/community involvement?

5. What would you change to improve the involvement processes?

6. In what ways did the final framework reflect consumer/carer/community needs?

7. In what ways was the final framework lacking?
